# An integrated neurophenomenological framework for naturalistic assessment of work-related stressors in healthcare professionals: a pilot study in neuro-surgery

**DOI:** 10.3389/fpsyg.2025.1568430

**Published:** 2025-04-28

**Authors:** Davide Crivelli, Elisa Pellencin, Alessandro Perin, Michela Balconi

**Affiliations:** ^1^International research center for Cognitive Applied Neuroscience (IrcCAN), Faculty of Psychology, Università Cattolica del Sacro Cuore, Milan, Italy; ^2^Department of Psychology, Università Cattolica del Sacro Cuore, Milan, Italy; ^3^Department of Neurosurgery, Fondazione I.R.C.C.S. Istituto Neurologico “C. Besta”, Milan, Italy; ^4^Besta NeuroSim Center, Fondazione I.R.C.C.S. Istituto Neurologico “C. Besta”, Milan, Italy

**Keywords:** work-related stressors, healthcare, neurosurgery, experience sampling, autonomic markers, wearables, neurophenomenology

## Abstract

Work-related stress and burnout are pervasive challenges in healthcare, particularly in high-stakes specialties like neurosurgery. Neurosurgeons face unique demands, including prolonged cognitive and physical strain, emotionally charged patient interactions, and ethical dilemmas. These stressors significantly impact individual wellbeing, patient safety, and organizational efficiency. However, traditional stress assessment methods, such as self-reports and retrospective surveys, fail to capture the dynamic and context-specific nature of stress in real-world clinical environments. This paper introduces an integrated neurophenomenological framework for assessing stress in neurosurgeons, combining continuous physiological monitoring with real-time phenomenological assessments using experience sampling methods (ESM). Wearable devices enable the collection of granular physiological data—heart rate, heart rate variability, electrodermal activity, and skin temperature—while ESM provides real-time subjective insights, reducing recall biases. Synchronizing these data streams offers a holistic understanding of stress dynamics. A pilot study is introduced to discuss the feasibility of this approach. Participants engaged in ward-based and surgical tasks while their physiological data were continuously recorded. Structured interviews and psychometric tools complemented these measures, revealing context-specific stress responses: higher electrodermal activity during emotionally demanding ward shifts and elevated heart rate during physically intense surgical procedures. Discrepancies between physiological activation and subjective stress perception highlighted the importance of interoceptive awareness in modulating stress responses. This framework offers a replicable model for advancing stress research in healthcare. By integrating physiological and phenomenological data, it provides actionable insights into stress dynamics, paving the way for targeted interventions to enhance resilience and optimize patient care.

## Work-related stress and burnout in healthcare: a focus on neurosurgery

Work-related stress and burnout represent pervasive challenges in the healthcare sector, with far-reaching consequences for both individuals and organizations. Neurosurgery, as a specialty, epitomizes a professional field where these challenges are particularly pronounced. Neurosurgeons work in environments characterized by exceptionally high stakes, requiring not only specific cognitive and technical skills but also the ability to sustain prolonged physical and psychological demands. The prevalence of burnout among healthcare professionals is well-documented, with studies estimating that between 30% and 60% of neurosurgeons experience symptoms of emotional exhaustion, depersonalization, and reduced professional accomplishment (Sinclair et al., 2017; Mackel et al., 2021; Barac et al., 2024). These conditions, which are key components of burnout, can result in diminished job satisfaction, reduced professional efficacy, and significant risks to patient care and safety.

The inherently demanding nature of neurosurgical practice exacerbates these risks (Neal and Lyons, 2020; Fernández-Villa de Rey-Salgado et al., 2024). Neurosurgeons frequently work extended hours, may endure sleep deprivation, and are exposed to high patient acuity and critical decision-making under pressure. Complex procedures, which require extraordinary precision and focus, can induce significant mental fatigue, while maintaining static, ergonomically strenuous postures over prolonged periods contributes to physical strain (Zulbaran-Rojas et al., 2024). Beyond these operational stressors, neurosurgeons often face profound moral and ethical dilemmas, particularly in decisions involving life-altering interventions or end-of-life care (Piquette et al., 2023; Chang et al., 2024). These cumulative stressors result in an occupational environment that can adversely affect individual wellbeing, job performance, and institutional outcomes. Elevated stress levels have been associated with increased medical errors, impaired communication, and compromised patient safety (Arora et al., 2010; Hénaux et al., 2019; Menon et al., 2020; Austin et al., 2021).

Stress in neurosurgery is both a psychological and physiological phenomenon, manifesting across multiple dimensions. Phenomenologically, it is experienced as emotional fatigue, moral distress, and a sense of being overwhelmed by external demands. These psychological responses are often magnified by systemic issues, including insufficient institutional support, understaffing, high workloads, and persistent conflicts between professional responsibilities and personal wellbeing (Neal and Lyons, 2020; Yust-Katz et al., 2020; Mackel et al., 2021; Barac et al., 2024; Fernández-Villa de Rey-Salgado et al., 2024). Physiologically, stress activates the autonomic nervous system, triggering measurable changes in biomarkers such as heart rate (HR), heart rate variability (HRV), electrodermal activity (EDA), and skin temperature. These markers reflect the body's attempts to adapt to stressors and offer objective indicators of acute and chronic stress responses (Weenk et al., 2018; Torkamani-Azar et al., 2022; Barac et al., 2024; Wang et al., 2024). As such, the integration of phenomenological insights with physiological measures is essential for comprehensively understanding how stress is perceived, experienced, and expressed in neurosurgical contexts.

On top of that, the consequences of stress and burnout may extend beyond the individual. At a personal level, chronic exposure to occupational stress increases the risk of mental health conditions, including anxiety, depression, and substance misuse (Oskrochi et al., 2016; Menon et al., 2020; Egbe and El Boghdady, 2024). Burnout can impair decision-making, attention, and executive functioning (Gavelin et al., 2022; Renaud and Lacroix, 2023)—key cognitive abilities that are critical for the safe and effective execution of neurosurgical procedures. For patients, the implications of neurosurgeon stress are profound. Elevated stress levels among surgeons have been linked to increased surgical complications, poorer patient outcomes, and heightened rates of medical errors (Arora et al., 2010; Hénaux et al., 2019; Menon et al., 2020; Austin et al., 2021). Furthermore, interpersonal dynamics within surgical teams are affected by stress, leading to impaired communication, reduced teamwork, and lower overall efficiency in the operating room (Hénaux et al., 2019; Fernández-Villa de Rey-Salgado et al., 2024).

At an organizational level, neurosurgeon burnout results in higher turnover rates, absenteeism, and diminished productivity, placing additional strain on already overstretched healthcare systems (Dewa et al., 2014; Shin et al., 2023). The financial implications are considerable, with burnout contributing to recruitment and training costs, as well as malpractice claims stemming from medical errors. These challenges underscore the urgent need for effective strategies to monitor and manage stress among neurosurgeons to protect both surgeon wellbeing and patient safety.

## Assessing work-related stress: state of the art and limitations

Traditional methods for assessing work-related stress—such as self-reports, retrospective surveys, and experience sampling methods (ESM; Dogan et al., 2022)—have provided valuable insights into individual experiences but come with inherent limitations. These methods are prone to recall bias and response distortions, and they lack the temporal resolution needed to capture the dynamic nature of stress in real-world clinical settings (Torkamani-Azar et al., 2022; Barac et al., 2024). For example, while widely used tools like the Maslach Burnout Inventory—MBI (Maslach and Jackson, 1981) or Perceived Stress Scale—PSS (Cohen et al., 1983) are effective for quantifying burnout and general stress levels, they fail to capture the moment-to-moment variability in stress experiences during the flow of everyday duties or high-demand tasks.

Recent advancements in wearable technologies have revolutionized the ability to monitor stress in real time by enabling non-invasive, continuous collection of physiological data in naturalistic settings. Devices such as smartwatches, ECG patches, and inertial measurement units (IMUs) can measure autonomic biomarkers, including HR, HRV, and EDA, offering granular insights into stress responses across the working day and during specific tasks (Weenk et al., 2018; Torkamani-Azar et al., 2022; Barac et al., 2024; Wang et al., 2024; Zulbaran-Rojas et al., 2024). For example, studies using wearable devices to monitor intensive care unit (ICU) residents demonstrated strong correlations between HR metrics and self-reported stress levels, underscoring the utility of such tools for identifying acute and chronic stress patterns (Wang et al., 2024). In neurosurgery, IMUs have also been employed to assess postural ergonomics during surgical procedures, providing actionable feedback to mitigate physical strain and prevent musculoskeletal injuries (Zulbaran-Rojas et al., 2024). These findings highlight the promise of wearable technologies in advancing stress monitoring, while also emphasizing their potential to enhance both individual wellbeing and organizational efficiency.

Despite their promise, the use of wearable technologies for professionals' stress monitoring in neurosurgery remains underexplored, and several challenges must be addressed for their integration into clinical practice. Ensuring data accuracy in dynamic, high-intensity environments like operating rooms is a significant hurdle, as motion artifacts and external interference can compromise signal quality. Moreover, the interpretation of multimodal datasets requires sophisticated analytical frameworks that can align physiological markers with subjective stress experiences in a meaningful way (Fairclough and Venables, 2006; Lahat et al., 2015; Booth et al., 2019). Privacy concerns related to the continuous collection of biometric data and the secure handling of sensitive information further complicate implementation. Nevertheless, wearable technologies represent a critical step toward overcoming the limitations of traditional stress assessments by providing objective, real-time insights that complement subjective data.

A critical limitation of the current literature on stress monitoring and assessment in healthcare—generally—and neurosurgery—more specifically—is the frequent focus on isolated dimensions of stress—either psychological or physiological—rather than on their integration. Most studies are conducted in controlled laboratory environments, which limits the ecological validity of findings and fails to reflect the complexity of real-world neurosurgical practice (Torkamani-Azar et al., 2022). Additionally, much of the existing research has been conducted in specific and selected international contexts, particularly the United States, where healthcare systems, cultural norms, and organizational structures present unique features. This discrepancy highlights the need for broader context-sensitive research efforts that account for the different stressors and coping mechanisms experienced by neurosurgeons within specific healthcare systems, capitalizing on such heterogeneity to identify both distinctive and shared criticalities. Furthermore, stress responses are highly individualized and influenced by professional experience. Junior neurosurgeons, such as residents and fellows, often experience greater levels of anxiety and moral distress due to their relative inexperience, higher workload intensity, and a perceived lack of autonomy. In contrast, senior professionals may exhibit more effective coping mechanisms, developed through years of practice, that buffer against the adverse effects of stress (e.g., Pascoe et al., 2021).

## An integrated neurophenomenological framework for naturalistic assessment of work-related stressors: preliminary remarks

Provided the above introduced state of the art and moving from such available evidence base, we posit that by integrating the potential of wearable neurotechnologies for sketching a granular view of stress-related autonomic changes and the qualitative resolution of ESM (Dogan et al., 2022), offering critical and rich insights into how stressors are perceived and experienced by individuals every day, it is possible to bridge the gap between subjective phenomenological data and objective physiological measures, fostering a more nuanced understanding of stress dynamics in healthcare settings.

The rationale for this integration is twofold. First, naturalistic assessments provide ecological validity, capturing the interplay of stressors as they unfold in the authentic environments where healthcare professionals operate. This is particularly important in neurosurgery, where stressors are shaped by task demands, team dynamics, and the emotional weight of patient outcomes. Second, combining ESM with wearable monitoring addresses the inherent limitations of each method. ESM mitigates the recall biases associated with traditional self-reports, while wearable devices provide an objective, continuous measure of physiological stress that can reveal patterns and fluctuations invisible to subjective methods alone.

To illustrate the feasibility and potential of this integrated approach, we here introduce a pilot study that applies such neurophenomenological protocol to a sample of neurosurgeons in a naturalistic clinical setting. The study aimed at demonstrating how wearable technologies and ESM can be used synergistically to map the physiological and phenomenological dimensions of stress, offering actionable insights into individual and contextual factors that shape stress responses. More specifically, the pilot aimed to test the integration of physiological monitoring and phenomenological self-report tools within real-world clinical settings and with respect to participants daily duties, to evaluate the coherence between subjectively perceived stress and objective biometric indicators, and to identify any logistical or methodological challenges requiring protocol refinement.

The study involved five neurosurgeons recruited from the Neurosurgical Oncology Department at the IRCCS Carlo Besta Neurological Institute in Milan. The sample consisted of two senior surgeons and three residents, ensuring variability in professional experience. The participants, with a mean age of 32.63 years (SD = 8.56), represented a balanced gender distribution, with three males and two females. This diversity facilitated an initial exploration of differences in stress experiences across professional levels, an essential consideration for the subsequent main study.

Data collection took place over two distinct workdays, paying attention to focus on equal working times (8 h on average in both days), with participants engaging in typical professional activities. The first workday focused on the operating room and related activities, while the second focused on ward and outpatient activities. On the first day, each participant was fitted with an Empatica E4 wristband, a wearable device capable of recording real-time physiological data such as heart rate (HR), heart rate variability (HRV), electrodermal activity (EDA), and skin temperature. The researcher provided initial instructions, secured informed consent, and ensured the device was correctly functioning before leaving the participant to proceed with their usual responsibilities. This departure was deliberate to minimize observer bias and maintain ecological validity. For the second day, participants independently donned the wearable device, demonstrating the ease and feasibility of the procedure.

Immediately following each shift, participants engaged in a structured interview designed to gather qualitative phenomenological data about their stress experiences. The interviews, lasting approximately 30 min, adhered to principles of ESM. Participants were guided through a reflective process to recall stressors encountered during the workday, along with their emotional, cognitive, and physiological responses. Specific attention was given to events that participants identified as particularly demanding, with probing questions exploring perceived control, coping strategies, and the intensity of stress experienced. These qualitative accounts were complemented by participants' evaluations of the overall shift, including levels of arousal, task-related demands, and general impressions of stress severity.

To further enrich the dataset, participants completed validated psychometric tools: the Perceived Stress Scale (PSS-10) to assess general perceived stress (Cohen et al., 1983), the Professional Quality of Life Scale (ProQOL-5) to measure burnout, compassion fatigue, and satisfaction (Stamm, 2010), and the Body Perception Questionnaire to evaluate interoceptive awareness and autonomic reactivity (Porges, 1993; specifically, we used the 22-item version of the tool by Poli et al., 2021). These tools provided a quantitative measure of stress, offering complementary insights to the physiological and interview data.

The preliminary outcomes of the pilot study highlighted several key findings. Physiological monitoring revealed context-specific variations in stress responses. Tonic levels of EDA, an indicator of sympathetic arousal (Dawson et al., 2007; Jarczok et al., 2013; Torkamani-Azar et al., 2022), was consistently elevated during ward shifts, suggesting the emotional and communicative demands of patient interactions as significant stressors. In contrast, heart rate (HR) and skin temperature increased during surgical procedures, reflecting the heightened physical and cognitive demands inherent to the operating room environment (Mestanik et al., 2015; Torkamani-Azar et al., 2022). HRV analyses indicated moderate autonomic activation, with variations depending on the complexity of tasks and situational stress.

The qualitative interviews identified consistent stressors across participants, with nuanced differences based on professional level. Ward shifts were characterized by emotional strain arising from delivering difficult diagnoses, managing patient families, and addressing high workloads. In the operating room, participants described stress linked to unpredictable complications, the need for precision under time constraints, and challenges associated with team dynamics. While all participants reported experiences of frustration, anxiety, and occasional feelings of being overwhelmed, they also highlighted moments of professional satisfaction, particularly following successful task completion. Interestingly, several participants noted a disconnect between their physiological states and conscious perceptions of stress, particularly during periods of sustained focus, underscoring the importance of interoceptive awareness in recognizing bodily responses to stress (Khalsa et al., 2018; Quadt et al., 2018).

Preliminary analyses demonstrated positive correlations between EDA peaks and participants' subjective stress ratings during specific events, affirming the utility of physiological biomarkers in detecting acute stress. Skin temperature, on the other hand, showed a negative association with stress, consistent with peripheral vasoconstriction during autonomic arousal. Notably, participants with higher scores on the BPQ-22 exhibited greater alignment between physiological markers and subjective stress reports, suggesting that interoceptive awareness may play a role in modulating stress perception (Mehling et al., 2011; Khalsa et al., 2018; Berntson and Khalsa, 2021; Poli et al., 2021).

The feasibility of the protocol was strongly supported by the pilot results. Participants were able to adhere to the wearable device usage with minimal disruption to their workflow, demonstrating the practicality of the physiological monitoring procedure. However, some challenges were identified, including motion artifacts in the data during highly dynamic tasks and logistical constraints related to conducting interviews immediately after shifts. These insights hint at potential minor adjustments to the protocol, such as optimizing artifact processing techniques and providing flexibility for interview scheduling to minimize participant burden.

Overall, the pilot study depose in favor of the robustness and ecological validity of the proposed experimental protocol, highlighting its ability to map occupational stress in a sample of neurosurgeons and in a naturalistic setting through a multi-method approach. The integration of objective physiological data, subjective self-reports, and qualitative insights proved effective for identifying both universal and context-specific stressors. Preliminary findings underscore the feasibility of implementing the protocol in larger cohorts. Furthermore, the pilot study revealed critical insights into the relationship between perceived and physiological stress responses, hinting at the need for interventions that enhance interoceptive awareness and provide tailored strategies for stress management (e.g., Balconi et al., 2017, 2019, 2020; Crivelli et al., 2019a,b). These preliminary outcomes may represent a solid foundation for next studies, offering a refined, validated framework to try and investigate occupational stress among neurosurgeons in real-world settings.

Nonetheless, it also has to be acknowledged that a robust and ecologically valid stress monitoring protocol must incorporate comparative analyses to fully capture the complexity of occupational stress responses in neurosurgical practice. Stress dynamics are likely to vary not only across different work contexts—such as spinal vs. cranial surgeries, outpatient consultations vs. acute care situations—but also according to task complexity, time pressure, and the degree of perceived responsibility. These factors may elicit distinct patterns of physiological activation and subjective distress, requiring context-specific analysis to disentangle their relative contributions. Moreover, individual differences play a critical role in shaping how stress is experienced and regulated. Professional seniority, for instance, might be associated with more adaptive coping mechanisms and greater emotional detachment in high-pressure scenarios, while junior professionals might exhibit heightened vulnerability due to lesser experience, reduced autonomy, and steeper learning curves. However, additional inter-individual factors such as age, baseline mental health status, and pre-existing physical conditions must also be considered as potential confounders. Age-related changes in autonomic reactivity and cognitive control could influence both physiological responses to stress and the accuracy of self-reported stress experiences. Similarly, undiagnosed or unreported psychological conditions—such as anxiety, depression, or burnout symptoms—can significantly alter stress sensitivity and recovery trajectories, potentially biasing physiological and phenomenological measures. In this light, future research would benefit from systematically controlling for these variables and integrating health status assessments into the study design. This would enhance the interpretability of stress profiles and improve the specificity of tailored interventions. Additionally, implementing feedback mechanisms based on real-time monitoring could empower neurosurgeons by fostering greater self-awareness and promoting proactive strategies to mitigate the impact of stress. These tools could be particularly valuable when combined with ergonomic training or resilience-building programs, ultimately supporting both individual wellbeing and clinical performance.

## Conclusions

The implications of naturalistic research dedicated to qualifying and quantifying primary causes and key subjective and objective correlates of stress responses in everyday life of neurosurgeons extend beyond the individual level. By reducing stress-induced errors, improving decision-making, and enhancing team communication, stress management strategies can directly contribute to patient safety and care quality. On an organizational level, the findings can inform the development of evidence-based wellbeing prevention and promotion programs, training initiatives, and systemic interventions that address institutional factors contributing to occupational stress. Moreover, the proposed multimodal framework—which integrates objective physiological monitoring, real-time phenomenological assessment, and qualitative interviews—offers a replicable model for investigating stress in other high-stakes medical and professional environments, fostering a broader understanding of work-related stress and its mitigation.

In conclusion, while wearable technologies and intensive experience sampling methods represent significant advancements in stress research, their application in neurosurgery is currently still limited. Addressing the methodological and practical challenges associated with these tools is essential for advancing our understanding of occupational stress in this unique context. The development and implementation of a comprehensive, ecologically valid monitoring protocol will not only bridge critical gaps in the existing literature but also pave the way for innovative strategies to enhance individual resilience, optimize team performance, and ensure the highest standards of patient care.

## Data Availability

The original contributions presented in the study are included in the article/supplementary material, further inquiries can be directed to the corresponding author.
